# Differential Responses of Neuronal and Spermatogenic Cells to the Doppel Cytotoxicity

**DOI:** 10.1371/journal.pone.0082130

**Published:** 2013-12-10

**Authors:** Kefeng Qin, Tianbing Ding, Yi Xiao, Wenyu Ma, Zhen Wang, Jimin Gao, Lili Zhao

**Affiliations:** 1 Zhejiang Provincial Key Laboratory for Technology and Application of Model Organisms, Wenzhou Medical University, Wenzhou, China; 2 Department of Microbiology, Fourth Military Medical University, Xi'an, China; 3 Department of Neurology, University of Chicago, Chicago, Illinois, United States of America; Van Andel Institute, United States of America

## Abstract

Although structurally and biochemically similar to the cellular prion (PrP^C^), doppel (Dpl) is unique in its biological functions. There are no reports about any neurodegenerative diseases induced by Dpl. However the artificial expression of Dpl in the PrP-deficient mouse brain causes ataxia with Purkinje cell death. Abundant Dpl proteins have been found in testis and depletion of the Dpl gene (*Prnd*) causes male infertility. Therefore, we hypothesize different regulations of *Prnd* in the nerve and male productive systems. In this study, by electrophoretic mobility shift assays we have determined that two different sets of transcription factors are involved in regulation of the *Prnd* promoter in mouse neuronal N2a and GC-1 spermatogenic (spg) cells, *i.e*., upstream stimulatory factors (USF) in both cells, Brn-3 and Sp1 in GC-1 spg cells, and Sp3 in N2a cells, leading to the expression of Dpl in GC-1 spg but not in N2a cells. We have further defined that, in N2a cells, Dpl induces oxidative stress and apoptosis, which stimulate ataxia-telangiectasia mutated (ATM)-modulating bindings of transcription factors, p53 and p21, to *Prnp* promoter, resulting the PrP^C^ elevation for counteraction of the Dpl cytotoxicity; in contrast, in GC-1 spg cells, phosphorylation of p21 and N-terminal truncated PrP may play roles in the control of Dpl-induced apoptosis, which may benefit the physiological function of Dpl in the male reproduction system.

## Introduction

Doppel (Dpl), Shaddo (Sho) and prion (PrP) belong to the prion protein family [1]. Although there are structural and biochemical similarities between Dpl and cellular prion (PrP^C^) [2]–[5], increasing lines of evidence suggest little similarities in functionalities between these two proteins [1], [6], [7]. In contrast, there is an active and antagonistic interaction between PrP^C^ and Dpl [3]. Dpl was initially identified as a homologue of PrP^C^ [2], [3]. The Dpl gene, *PRND* or *Prnd*, has been identified in a wide range of vertebrates, including fishes, tetrapods [8]–[10], cattle, sheep [11], goat [12], mouse and human [2], [3], [13], suggesting that Dpl is a highly conserved cellular protein. Nevertheless, little is known at present about the normally physiological function of Dpl. Dpl apparently has two biological effects, *i.e*., it is toxic in the central nerve system if it is artificially expressed when PrP^C^ is absent [3] but it is needed in the male fertility [14], [15].

Dpl binds copper ions, but the physiological relevance of this copper binding has not yet been clearly defined [5], [16]–[18]. The overexpression of *Prnd*, especially in PrP^C^-knockout (PrP^0/0^) mice, causes progressive ataxia with the Purkinje cell loss in cerebellar folia [3], [19], [20] and demyelination of peripheral nerves [21], suggesting that Dpl, in the absence of PrP^C^, induces neuropathogenic damages mimicking neurodegeneration, which is different from prion diseases. Our previous studies have further shown that Dpl induces apoptosis in neuronal N2a cells through a mitochondrion-independent mechanism leading to caspase-10 and caspase-3 cleavages [6]. A mutagenesis study of *Prnd* indicates that the αB/B′-loop-αC region is a core determinant for Dpl-induced apoptosis [22]. Some lines of evidence have also shown that Dpl directly interacts with PrP^C^ [6], [23]. In contrary to the cytotoxic effect of Dpl in neuronal tissues, the high level of Dpl in spermatogenic cells does not appear to have any obvious toxic effect. In fact, Dpl plays an important role in sexual differentiation, especially in spermatogenesis [24], [25]. Indeed, the activity of Dpl is required for the male reproduction because the Dpl-deficient male mice are sterile [14]. The spermatozoa isolated from Dpl-knockout mice showed several structural abnormalities and were unable to fertilize wild type oocytes [14], [15]. Further examination of those abnormal spermatozoa have revealed that Dpl is a critical regulator of spermatogenesis and the acrosome reaction, *i.e*., sperm-egg interaction [14], [15]. Interestingly, recombinant Dpl enhances the ovine spermatozoa fertilizing ability [26].

The expression of *Prnd* is highly tissue-specific and also developmental stage-dependent within the same type of cells. The Dpl protein level is normally very low or undetectable in the adult brain but is highly abundant in male germ line cells [3], [4], [27], suggesting that the *Prnd* expression might be differentially regulated in differently cellular environments. Even though little or no Dpl protein is detected in adult neuronal cells, relatively high level of Dpl can be detected in embryonic neurons such as dorsal root ganglia [28] and brains of new-born mice [29]. Furthermore, different expression patterns of Dpl have also been detected in germinal cells [24], [25], indicating a possible role of Dpl in germinal cell differentiation. In cell culture models, Dpl is abundant in the reproductive cellular lineage such as GC-1 spermatogenic (spg) cells [30] but little in the neuronal cellular lineage such as N2a cells [3], [6]. Therefore, certain cell lines such as the neuronal lineage are prone to apoptosis induced by Dpl [6], [22], but others such as spermatogenic cell lines should be resistant to Dpl-induced apoptosis. However, molecular mechanism underlying susceptivity and resistance of these cells to Dpl-induced apoptosis is currently unknown.

In the present study, we have questioned whether different sets of regulatory molecules involve in the expression of Dpl and in the response to the Dpl-induced apoptosis in neuronal and spermatogenic cells. To answer the question, we used N2a and GC-1 spg cells as cell culture models. We have shown that the Dpl expressions in N2a and GC-1 spg cells are regulated at the transcriptional level by two sets of transcription factors. In N2a cells, Dpl-induced PrP^C^ elevation is through ATM-modulating transcription regulation. In addition, different forms of PrP^C^ may play roles in responses to Dpl toxicity in these pro- and anti-apoptotic cells.

## Materials and Methods

### Monoclonal antibody against Dpl

The hybridoma cell line secreting monoclonal antibody (mAb) 1A9 to doppel was established by fusing mouse myloma cell line Sp2/0 with spleen cells of BALA/c mice immunized by the purified and refolded recombinant mouse Dpl protein [4], [5], [31]. IsoStrip in Mouse Monoclonal Antibody Isotyping Kit (Roche, Basel, Switzerland) was used to determine the mAb subtype. The target epitope of mAb 1A9 was identified by using Pepscan technique (Pepscan System BV, Lelystad, Netherlands).

### Cell culture, transfection and Dpl treatment

Murine neuro-2a (N2a) and GC-1 spermatogenic (spg) cells were cultured in Dulbecco's modified Eagle medium (DMEM) supplemented with 10% fetal bovine serum (FBS) (Invitogen, Carlsbad, CA, USA) at 37°C and 5% CO_2_. To express the plasmid-driven Dpl, pcDNA3-Dpl or the vector pcDNA3 was transfected into N2a cells in a 10 cm-plate with 24 µg of plasmid DNA using lipofectamine 2000 (Invitrogen) following the manufacturer's instruction. To knock down the ATM expression, we used the pre-designed small interfering RNA (siRNA) to ATM (Ambion, Austin, TX, USA) and the control non-silencing siRNA (Qiagen, Valencia, CA, USA). Each siRNA at a concentration of 100 nM was transfected into ∼5×10^5^ N2a cells using 15 µl of Oligofectamine following the manufacturer's instructions (Invitrogen). In some cases, cells were incubated with or without 20 µg/ml of the purified recombinant Dpl protein [4], [5], [31] for 0, 1, 2 or 4 h and then harvested for further experiments.

### Luciferase assays

N2a or GC-1 spg cells in 6-well plates were co-transfected with 2 µg of pTAL-Luc reporter vector (a gift from Dr. Kopacek) and 100 ng of pRL-TK renilla vector (Promega, Madison, WI, USA) that served for the control of the transfection efficiency using Lipofectamin 2000 (Invitrogen) according to the manufacturer's recommendation. The expressions of different reporter genes were assessed using the Dual-Luciferase Reporter Assay System (Promega) 72 h after transfection. For comparison, the profile of the luciferase activity produced by different constructs was normalized against the renilla expression.

### Measurement of ROS

N2a and GC-1 spg cells in 96-well plates were incubated with 50 µM 2′,7′-dichlorodihydrofluorescein diacetate (DCFH-DA) (Invitrogen) for 45 min for DCFH-DA to diffuse into cells. After wash with phosphate-buffered saline (PBS), cells were then treated with 20 µg/ml Dpl, or 100 µM CuCl_2_ as a positive control and 100 µM MgCl_2_ as a negative control, for 0, 1, 2 or 4 h. DCFH-DA is hydrolyzed by intracellular esterase to yield DCFH that is oxidized by H_2_O_2_ or low-molecular weight peroxides in cells to produce the highly fluorescent compound, 2′, 7′-dichlorofluorescein (DCF). After wash with PBS, the DCF fluorescence intensity is determined using a Multiable Counter (Model Wallac 1420, Perkin Elmer, Waltham, MA, USA) with an excitation wavelength of 485 nm and an emission wavelength 530 nm.

### Measurement of cell viability

The cell viability was measured by a 3-[4,5-dimethylthiazol-2-yl]-2,5-diphenyl tetrazolium bromide (MTT) based cell growth determination kit (Sigma-Aldrich, St. Louis, MO, USA). N2a or GC-1 spg cells were incubated with 20 µg/ml Dpl, or 100 µM CuCl_2_ as a positive control and 100 µM MgCl_2_ as a negative control, for 0, 1, 2 or 4 h. After removed medium, cells were aseptically added with MTT solution in an amount equal to 10% of the culture volume and incubated for 4 h. MTT formazan crystals were dissolved by addition of MTT solvent in an amount equal to the original culture volume and incubation for 1 h. Absorbance was spectrophotometrically measured at a wavelength of 570 nm with subtraction of background absorbance measured at 690 nm.

### Western blot analyses

Cells were homogenized in a lysis buffer, 50 mM Tris–HCl, pH 7.5, 150 mM NaCl, 2 mM EDTA, 1% Triton X-100, containing a protease inhibitor cocktail (Roche), on ice for 30 min. After centrifugation at 10,000 r.p.m. for 10 min, the supernatant was collected for experiments. After determination of protein concentrations by BCA protein assay (PIERCE, Woburn, MA, USA), the equivalent of 30 µg of total protein was loaded onto SDS-PAGE gels (Bio-Rad, Hercules, CA, USA) and analyzed by Western blotting with the appropriate primary antibodies, *i.e.*, 1∶500 dilution of anti-Dpl mAb 1A9 or anti-PrP mAb SAF-32 (Cayman Chemical, Ann Arbor, MI, USA), 1∶1,000 dilution of antibody against phosphorylated p53-Ser15 or p21 (Cell Signaling, Beverly, MA, USA), 1∶500 dilution of anti-Sp1 antibody (Santa Cruz Biotech, Santa Cruz, CA, USA), 1∶10,000 dilution of anti-β-actin antibody (Sigma-Aldrich). The appropriate horseradish peroxidase (HRP)-conjugated secondary antibodies (Bio-Rad) were used. The blots were visualized by using ECL kit (Amersham, Uppsala, Sweden) and exposed in Image Universal Hood II (Bio-Rad).

### Electrophoretic mobility shift assays (EMSA)

Two sets of cells were cultured for nuclear protein extracts. N2a and GC-1 spg cells were used as the first set of cells. As the second set, N2a cells were mock-transfected or transfected with control siRNA or siRNA to ATM for 48 h and then incubated with or without 20 µg/ml Dpl for 2 h. Harvested cells were gently resuspended in Lysis Buffer A, 10 mM Hepes, pH 8, 1.5 mM MgCl_2_, 10 mM KCl, 0.3 M Sucrose, 1 mM DTT, 0.5% NP-40, containing a protease inhibitor cocktail (Roche), and then incubated on ice for 15 min. After centrifugation at 800 r.p.m. for 5 min, the cell pellet were resuspended in Lysis Buffer B, 10 mM Hepes, pH 8, 25% Glycerol, 0.42 M NaCl, 1.5 mM EDTA, containing a protease inhibitor cocktail (Roche), and then rocked at 4°C for 2 h. After centrifugation at 14,000 r.p.m. for 10 min, the supernatants were stored as the nuclear protein extracts and ready for EMSA. The linear DNA fragments containing DNA sequences of the *Prnd* promoter, *i.e*., −1295/−1278 and −1215/−1199 (A and T rich regions), −191/−167 (E-box), and −57/−28 (GC-box), and in the mouse *Prnp* promoter region (−1928/+52) [32] were used as target DNAs. The nucleotide sequences of the probe sense strands are as follows: the putative Sp1 binding sites (−65/−35): 5′-ATC ACG CCC CGC CCC TCG CCC AGC CTA GCT CC-3′ and the putative p53 binding site (−1832/−1810): 5′-CTT GTC AAG ACT AGT TTG CCT CG-3′ in the mouse *Prnp* promoter. As competitors, the DNA fragments also include: the Sp1 consensus 5′-ATT CGA TCG GGG CGG GGC GAG C-3′ [33] and the p53 consensus: 5′-AGG CAT GTC TAG GCA TG-3′ [34]. Target DNAs were labeled with biotin by Biotin 3′-End DNA Labeling kit (PIERCE). Two complement DNA sequences of the biotin-labeled target DNAs or unlabeled (cold) consensuses were annealed to be double strand oligonucleartides. LightShift Chemiluminescent EMSA Kit (PIERCE) were used in the DNA-protein binding experiments. The biotin-labeled double strand target DNA was incubated with the nuclear extract from cells in the condition of 2.5% glycerol, 5 mM MgCl_2_, 1 mM EDTA, 0.05% NP-40 and 50 ng/µl Poly(dI⋅dC) at room temperature for 20 min, and then load onto 5% polyacylamide gel in 0.5× TBE for electrophoresis. The binding reaction will be transfer to a Nylon membrane (PIERCE). After cross-linking the transferred DNA to the membrane by ultraviolet (UV) light, the biotin-labeled DNA signals were detected by sequential incubations of the membrane with stabilized strptavidin-HRP conjugate and lumino/enhancer solution (PIERCE), and then expose of the membrane to X-ray film. The binding of the predicted transcription factor and the specific target DNA formed a slower-migrated band that shifts from the band of the starting target DNA. If a shift band has been seen in EMSA, it is an indication that one of predicted transcription factors binds to a specific DNA sequence in the promoter of the mouse *Prnd* or *Prnp*. At this time, 100-fold molar excess of the unlabeled consensus was mixed with the biotin-labeled starting target DNA and then EMSA were performed.

## Results

### Expressions of doppel and prion in neuronal N2a and GC-1 spermatogenic cells

**Figure 1:**
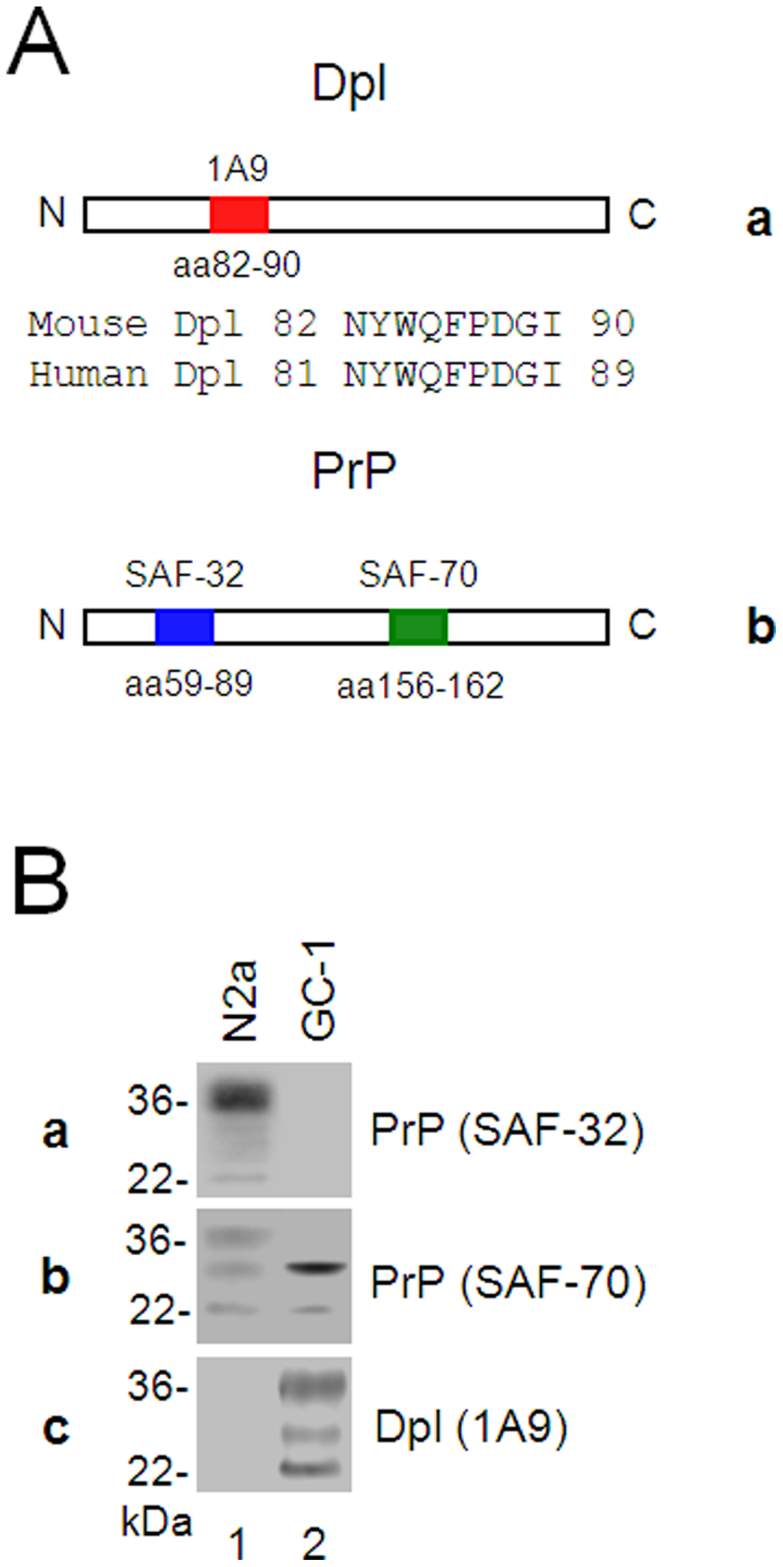
Dpl and PrP are differentially expressed in N2a and GC-1 spermatogenic cells. A. The target epitope of monoclonal antibody 1A9 to Doppel (a) and SAF-32 or SAF-70 to PrP (b). B. Expressions of Dpl and PrP in N2a and GC-1 spg cells. Cell lysates of mouse N2a (lanes 1) or GC-1 spg cells (lanes 2) were subjected to Western blots detecting by anti-PrP mAb SAF-32 (a), SAF-70 (b), or anti-Dpl mAb 1A9 (c).

The Hybridoma line 1A9 was derived by fusing Sp2/0-A9-14 myeloma cells to spleen cells from BALB/c mice immunized with the purified mouse Dpl protein. The monoclonal antibody (mAb) 1A9 belongs to IgG1 subtype with κ light chains determined by Isotyping Kit. By using Pepscan technique the target epitope of mAb 1A9 is identified between amino acid (aa) residues 82–90 (NYWQFPDGI) that is 100% identical to human Dpl sequence aa81–89 (Fig. 1A, a). Using mAb 1A9, we had detected the Dpl expression in the mouse GC-1 spermatogenic (spg) cells (Fig. 1B, c2) but not in N2a cells (Fig. 1B, c1), confirming the specificity of mAb 1A9 to Dpl. By using mAb SAF-32 recognizing N-terminal aa59–89 and mAb SAF-70 recognizing C-terminal aa156–162 (Fig. 1A, b), we have detected the PrP expression in full-length with non-, mono-, and di-glycosylation in N2a cells (Fig. 1, B, a1, b1), but in an N-terminal truncated form in GC-1 spg cells. These phenomena may indicate that two PrP forms might have their own functions in different types of cells.

### The *Prnd* promoter is activated at a high level in GC-1 spg cells but at a low level in N2a cells

**Figure 2:**
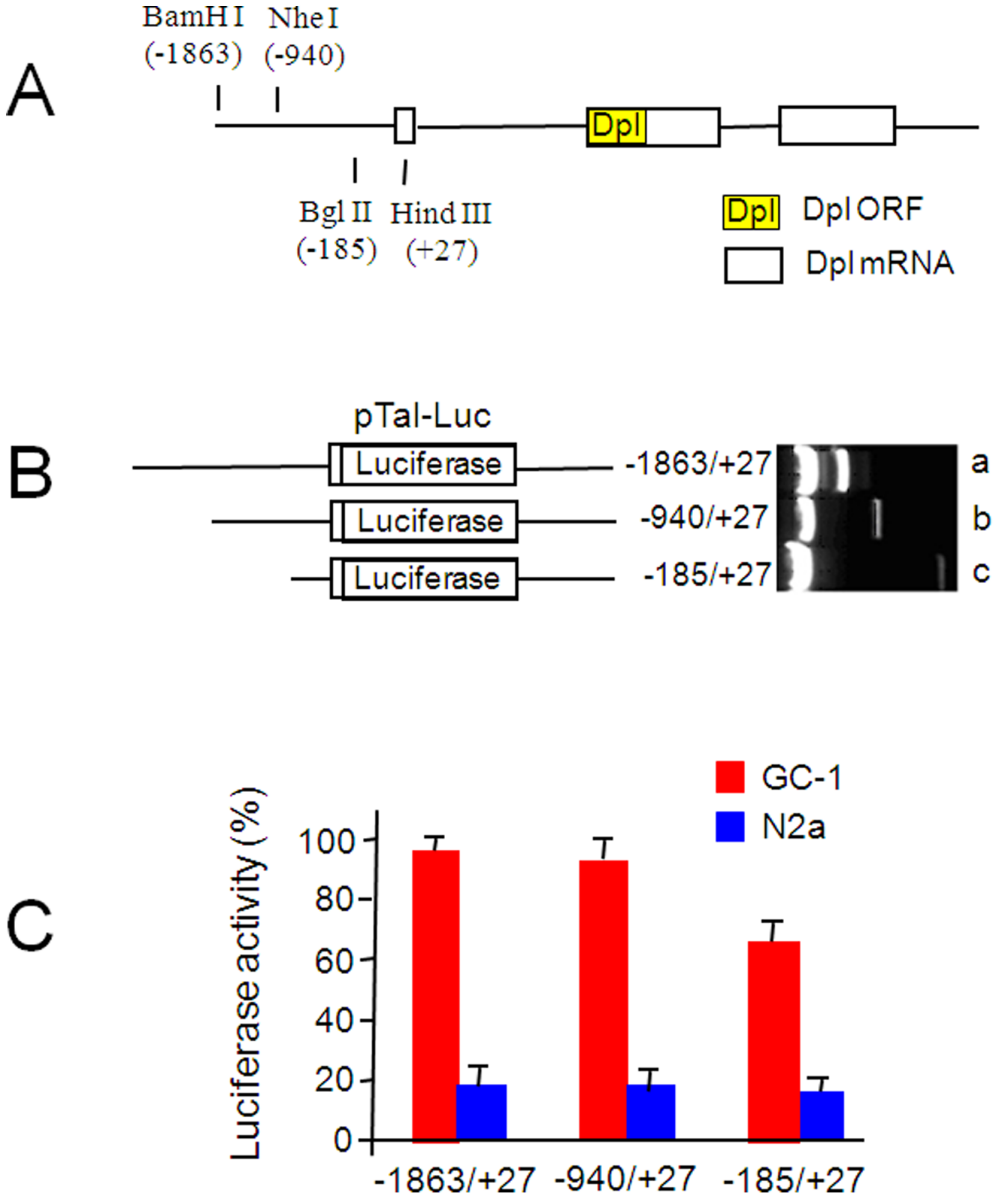
The differential activates of *Prnd* promoter are measured in N2a and GC-1 spg cells. A. Schematic structure of the *Prnd* and its promoter construct: 5′-flanking region and exon-intron organization of the *Prnd* region. The numbers of restriction sites represent the distances from the mRNA start site. B. The constructs of the *Prnd* promoter in pTal-Luc reporter vector. The plasmid containing the *Prnd* promoter regions of −1863/+27, −940/+27 or −185/+27 was digested with BamH I+Hind III (a), Nhe I+Hind III (b) and Bgl II+Hind III (c), respectively, and then run on the 1% agarose gel. **C**. Transcriptional activities of the *Prnd* promoter. N2a and GC-1 spg cells were transfected with the different *Prnd* promoter constructs (B) in pTal-Luc vector. Promoter activities are expressed in % relative to the activity of the full *Prnd* promoter construct (−1863/+27) in GC-1 spg cells, which was set to 100%. Bars are means ± SD of 3 independent experiments. Student *t* test was used for statistic analyses.

To determine whether the Dpl expression is differently regulated in two types of cell lines, we have firstly made the fine mapping of the *Prnd* promoter. The mouse *Prnd* promoter has been mapped to the region −1863/+27 of the mRNA start site (Fig. 2A) [30]. To determine the transcriptional activity of the *Prnd* promoter in N2a and GC-1 spg cells, we used a modified pTal-Luc reporter vector, in which the firefly luciferase gene was fused with different DNA regions of the *Prnd* promoter (Fig. 2B). The pRL-TK vector that contains a HSV TK promoter driving a Renilla luciferase gene was used as a transfection efficiency control. Expressions of both Luc reporter genes were measured using the Dual-Luciferase Reporter Assay System 72 h after transfection. Similar amounts of Renilla luciferase were detected in N2a and GC-1 spg cells (data not shown). As shown in Fig. 2C, high levels of the firefly luciferase activities were detected in GC-1 spg cells, *i.e*., −1863/+27: 100±4.7%, −940/+27: 94.2±11.4% and −185/+27: 67.7±6.9%, but relatively low levels in N2a cells, *i.e*., −1863/+27: 20.4±5.4%, −940/+27: 18.5±4.5% and −185/+27: 17.9±3.9%. These results suggest that transcriptional regulations of the *Prnd* promoter regions are positive in GC-1 spg cells but relatively negative in N2a cells.

### Different transcription factors bind to the *Prnd* promoter in N2a and GC-1 spg cells

**Figure 3:**
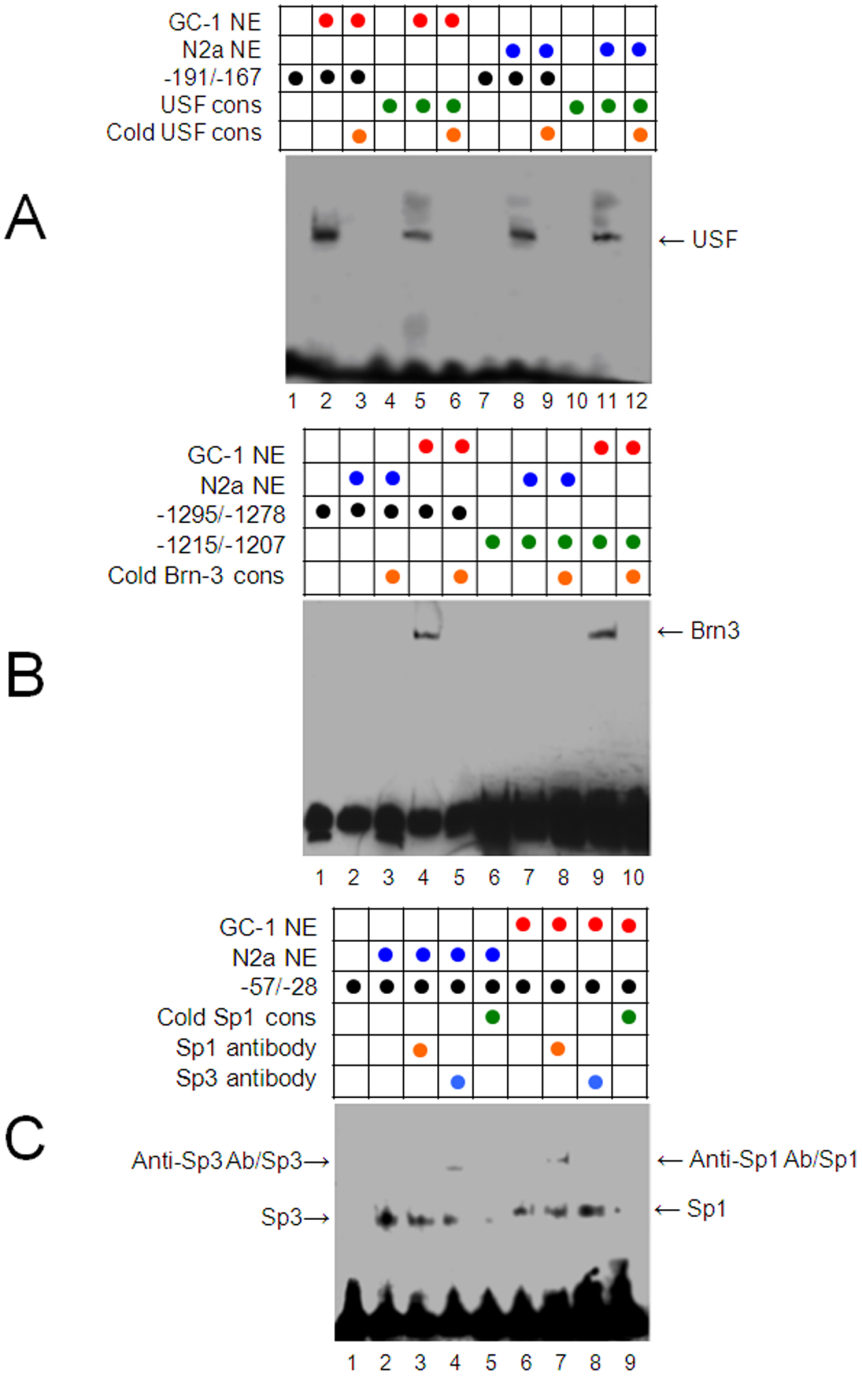
Different sets of transcription factors bind to *Prnd* promoter in N2a and GC-1 spg cells determined by gel-shift analyses with the nuclear extract (NE) from GC-1 spg and N2a cells. A. USF binds to E-box in both N2a and GC-1 cells; B. Brn-3 binds to the G and T rich regions −1295/−1278 and −1215/−1199 in GC-1 spg but not in N2a cells; C. Sp1 binds to GC-box in GC-1 cells and Sp3 binds to GC-box in N2a cells. Combination of the oligonucleotides and nuclear extracts used in assays are indicated by colorful circles above the radiogram.

To further understand the transcriptional regulation of the *Prnd* promoter, we used the electrophoretic mobility shift assay (EMSA) to identify regulatory transcription factors those bind to the mouse *Prnd* promoter. DNA sequences, *i.e*., −1295/−1278 and −1215/−1199 (A and T rich regions), −191/−167 (E-box), and −57/−28 (GC-box), in the *Prnd* promoter, were labeled with biotin at their 3′-ends, and then incubated with nuclear extracts isolated from N2a or GC-1 spg cells. DNA-protein binding complexes were resolved on a 5% TBE native gel (Fig. 3). The transcription factors those may regulate the Dpl expression are described below:

a) Upstream stimulatory factors (USF). Consistent with an early report [35], incubation of the biotin-labeled E-box (−191/−167) or USF consensus with nuclear extracts from GC-1 spg cells formed a major DNA-protein binding band (Fig. 3, A2, A5). Addition of 100-fold molar excess cold USF consensus completely blocked the binding of proteins to either E-box or USF consensus (Fig. 3, A3, A6). A similar DNA-protein binding profile was also found in N2a cells (Fig. 3, A8, A11), and addition of 100-fold molar excess cold USF consensus also completely blocked the binding of proteins to either E-box or USF consensus (Fig. 3, A9, A12). These findings suggest that the same transcription factor USF (USF-1 or USF-2) binds to E-box of the *Prnd* promoter both in GC-1 spg and N2a cells.

b) Brn-3. It is reported that the Dpl mRNA level was decreased in the brain of the Brn-3a knockout mice and transfection of human neuroblastoma ND7 cells with Brn-3a or Brn-3b containing plasmids caused the increase in Dpl protein levels [28]. Thus, we made the effort to define the possible Brn-3 binding sites in the *Prnd* promoter. The DNA fragments −1295/1278 or −1215/-1199 contains the sequence similar to the conserved binding site for Brn-3 of POU IV class. Incubation of each of these DNA fragments with nuclear extracts from N2a cells resulted no DNA-protein binding bands (Fig. 3, B2, B3, B7, B8). In contrast, incubation of the DNA fragment −1295/1278 or −1215/−1199 with nuclear extracts from GC-1 spg cells resulted in a band representing the DNA-protein binding complex (Fig. 3, B4, B9). This band could be polished by 100-fold molar exceed cold Brn-3 consensus (Fig. 3, B5, B10). Therefore, this is the first evidence that Brn-3 (Brn-3a or Brn-3b) does bind to the *Prnd* promoter in GC-1 spg but not in N2a cells. Since Brn-3 is a positive transcription factor, this novel finding may provide an explanation why Dpl is expressed at a high level in GC-1 spg cells but at an undetectable level in N2a cells.

c) Sp1-like family. Sp1-like protein family contains four members: Sp1, Sp2, Sp3 and Sp4. A computer search of the *Prnd* promoter sequence revealed a putative Sp1/Sp3 binding site, GC-box, which resides between positions of −58/−27 in the *Prnd* promoter. To test whether the Sp1-like transcription factors bind to the *Prnd* promoter, the biotin-labeled sequence −58/−27 was incubated with the nuclear extracts from N2a or GC-1 spg cells. One DNA-protein binding band was detected in both N2a and GC-1 spg cells (Fig. 3, C2, C6). Addition of 100 molar more of the cold Sp consensus sequence [33] near completely abolished this DNA-protein binding band (Fig. 3, C5, C9), suggesting the Sp1-like proteins indeed bind to the *Prnd* promoter. Since both Sp1 and Sp3 bind to the same Sp consensus sequence, we performed supergelshift experiments to further distinguish these two transcription factors. Addition of anti-Sp3 antibody, but not anti-Sp1 antibody, to the GC-box/N2a nuclear extract results an additional slower migrating band (Fig. 3, C4 vs. C3). Thus, this band corresponds to the binding of antibody/Sp3/GC-box DNA fragment. In contrast, addition of anti-Sp1 antibody, but not anti-Sp3 antibody, to the GC-box/GC-1 nuclear extract results a band corresponding to the binding of antibody/Sp3/GC-box DNA fragment (Fig. 3, C7 vs. C8). These observations suggest that a differential binding of the same *Prnd* promoter region by Sp1 in GC-1 spg cells and by Sp3 in N2a cells. Interestingly, Sp1 is a known transcriptional activator [36], [37] but Sp3 can act either as a transcriptional activator or repressor [38]. For example, Sp3 can competes the GC-box binding with Sp1 resulting in negative regulation of the promoter [39]–[42].

### Dpl induces oxidative stress in N2a but not in GC-1 cells

**Figure 4:**
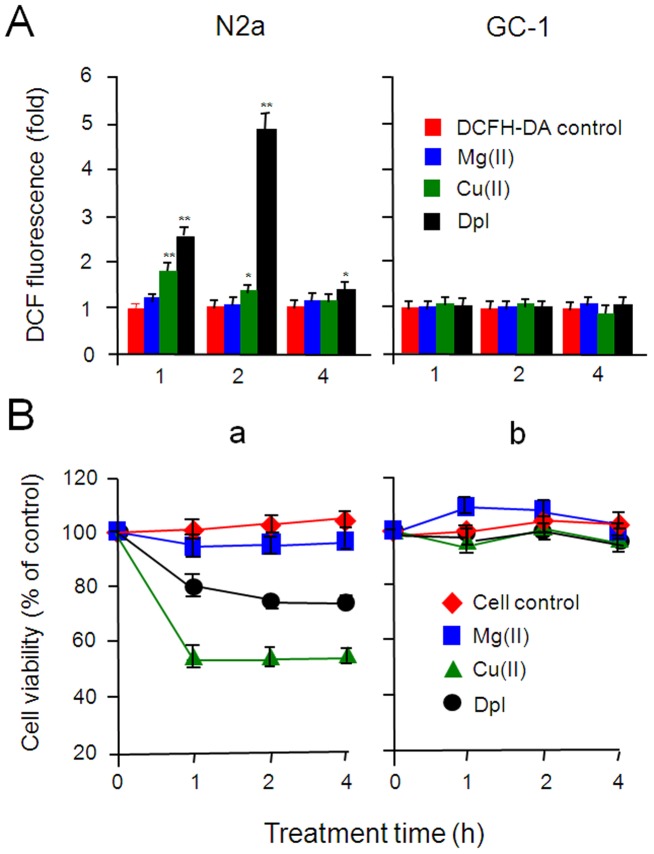
Dpl induces ROS accumulation and acute cell death in N2a but not in GC-1 spg cells. A. Dpl induces ROS accumulation. N2a (a) or GC-1 spg cells (b) in 96-well plates were incubated with 50 µM DCFH-DA for 45 min. After wash, cells were then incubated without or with MgCl_2_ (100 µM), CuCl_2_ (100 µM) or the purified mouse Dpl protein (20 µg/ml) for 1, 2 or 4 h. After wash, DCF fluorescence was determined at an excitation of 485 nm and emission of 538 nm by a microplate-reader. The readings of DCF fluorescence of each test group were normalized against that of the DCFH-DA control group and expressed as relative-fold change. Bars are means ± SD of 3 independent experiments. Student *t* test was used for statistic analyses (*, *p*<0.05; **, *p*<0.01). B. Dpl induces acute cell death. N2a (a) or GC-1 spg cells (b) in 96-well plates were incubated without or with MgCl_2_ (100 µM), CuCl_2_ (100 µM), or the purified Dpl (20 µg/ml) for 0, 1, 2, or 4 h. The cell growth curves were determined by MTT assay. Bars are means ± SD of 3 independent experiments. Student *t* test was used for statistic analyses.

To determine whether Dpl induces the oxidative stress in N2a and GC-1 spg cells, we used the 2′,7′-dichlorodihydro-fluorescein diacetate (DCF-DA) assay to test the potential effect of Dpl on accumulation of reactive oxygen species (ROS). N2a and GC-1 spg cells were incubated with 20 µg/ml of the recombinant Dpl [4], 100 µM of Mn(II) or Cu(II) as negative or positive control [43]. As shown in Fig. 4A, a, in N2a cells, no significant increase of ROS could be observed after incubation with Mn(II) for 1, 2 and 4 h; the rapid accumulation of ROS was induced by Cu(II) in 1 h (1.85±0.05 fold, *p*<0.01) followed by declining in 2 h (1.23±0.12 fold, *p*<0.05) and in 4 h (1.18±0.10 fold, *p*<0.05). Similar to Cu(II), Dpl also caused rapid but higher levels of ROS in N2a cells in 1 h (2.58±0.12 fold, *p*<0.01) and in 2 h (4.86±0.28 fold, *p*<0.01) following declining in 4 h (1.49±0.16 fold, *p*<0.01). The rapid declines of ROS induced by Cu(II) and Dpl may in part be attributed to the presence of PrP^C^ [44]. Interestingly, no accumulation of ROS was detected in any of the treatments of GC-1 spg cells (Fig. 4A, b), suggesting that GC-1 spg cells are resistant to ROS induced by both Cu(II) and Dpl.

### Dpl induces cell death in N2a but not in GC-1 spg cells

To test whether ROS induced by Dpl leads to cell death, we examined the effects of Dpl, Cu(II) or Mn(II) on the cell viability by using the 3-[4,5-dimethylthiazol-2-yl]-2,5-diphenyl tetrazolium bromide (MTT) colorimetric assay [45]. Cells were incubated with 100 µM of Mg(II) or Cu(II), or the purified Dpl (20 µg/ml) for 1, 2 or 4 h. Cellular viability was calculated by MTT based on cell growth curves. As shown in Fig. 4B, Mg(II) did not induced both N2a and GC-1 spg cell death. Cu(II) induced the reduction in the cellular viability of 53.0±3.3%, 53.3±4.9%, and 54.4±2.0% at 1, 2, and 4 h, respectively, in N2a cells but no significant changes in GC-1 cells by comparing with that in untreated cells. Similar to Cu(II), Dpl induced significant reduction in the cellular viability of 79.1±13.7%, 74.5±3.6%, and 74.1±4.7% at 1, 2, and 4 h, respectively, in N2a cells,but no significant changes in GC-1 cells. By comparison of MTT results by Cu(II) and Dpl, Cu(II) induced more cell death than Dpl did, implying that beside the oxidative stress effect, Cu(II) has its own metal effects. Cu(II) and Dpl induce the cell viability reduction in N2a but not in GC-1 spg cells, indicating that GC-1 spg cells have mechanisms against oxidative stress caused by Cu(II) or Dpl.

### Dpl induces apoptosis in N2a but not in GC-1 cells

**Figure 5:**
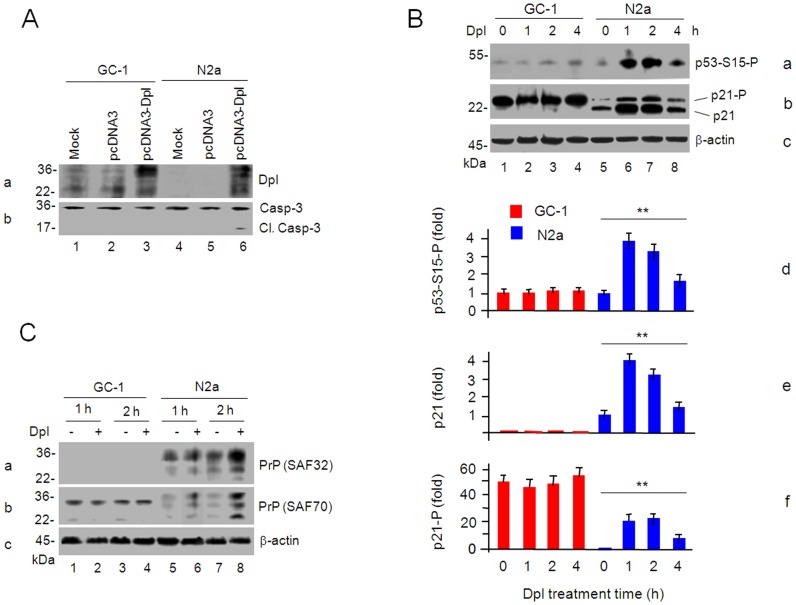
Dpl induces differently molecular effects in N2a and GC-1 cells. A. Dpl induces apoptosis in N2a cells but not in GC-1 spg cells. GC-1 spg (lanes 1-3) or N2a cells (lanes 4-6) were mock-transfected (lanes 1, 4) or transfected with pcDNA3 (lanes 2, 5) or pcDNA3-Dpl (lanes 3, 6) for 72 h. Cell lysates were subjected to Western blots with anti-Dpl mAb 1A9 (a) or anti-caspase-3 antibody (b). B. Dpl induces the phosphorylation of p53 and p21 in N2a but not in GC-1 spg cells. GC-1 spg (lanes 1–4) or N2a cells (lanes 5–8) were incubated with the purified Dpl protein (20 µg/ml) for 0, 1, 2 or 4 h. Cell lysates were subjected to Western blots with anti-phosphorylated p53 at S15 antibody (a), anti-p21 antibody (b) and anti-β-actin antibody (c). The protein signals of phosphorylated p53 at S15 (p53-S15-P) (a, d), p21 (b, e) and phosphorylated p21 (p21-P) (b, f) were scanned, normalized to β-actin levels (c), and expressed as relative -fold change over signals in untreated GC-1 spg cells (a1) or N2a cells (b5, c5). Bars represent the mean ± S.D. of three independent experiments. ANOVA were used for statistic analyses (**, *p*<0.01). C. Dpl induces the elevation of the full-lengh PrP^C^ in N2a cells but has no effect on the N-terminal truncated PrP^C^ in GC-1 spg cells. N2a (lanes 5–8) or GC-1 cells (lanes 1–4) were incubated without (lanes 1, 3, 5, 7) or with Dpl (20 µg/ml) (lanes 2, 4, 6, 8) for 1 h (lanes 1, 2, 5, 6) or 2 h (lanes 3, 4, 7, 8). Cell lysates were subjected to Western blots with anti-PrP mAb SAF-32 (a), SAF-70 (b), or anti-β-actin antibody (c).

We have previously shown that overexpression of Dpl in N2a cells and primary astrocytes induces apoptosis through caspase-3 cleavage [31]. To further compare cellular responses to Dpl-induced apoptosis, we overexpressed Dpl in N2a and GC-1 spg cells by transfection with pcDNA3 plasmids carrying the *Prnd* gene for 72 h and then measured caspase-3 cleavage. Consistent with prior reports that the high level expression of the Dpl mRNA in GC-1 spg cells [30], relatively high level of endogenous Dpl was detected in the GC-1 spg cells (Fig. 5A, a1) whereas no detectable Dpl was expressed in N2a cells (Fig. 5A, a4). Transfection with the vector plasmids pcDNA3 did not change Dpl expression patterns (Fig. 5A, a2, a5). Transfection with the *Prnd*-carrying plasmids resulted in similar levels of the Dpl in both types of cells (Fig. 5A, a3, a6). No cleaved caspase-3 was observed in either type of cells without overexpression of Dpl, even though relatively high level of Dpl was also detected in the GC-1 spg cells (Fig. 5A, b1–2). Significantly, overexpression of Dpl induced cleavage of caspase-3 in N2a cells (Fig. 5A, b6) but it did not cause any obvious cleavage in the GC-1 spg cells (Fig. 5A, b3), suggesting that Dpl causes apoptosis in N2a cells but not in GC-1 spg cells.

### Dpl induces phosphorylation of p53, elevation and phosphorylation of p21 in N2a but not GC-1 spg cells

To determine whether Dpl induces the signal transduction, we incubated N2a and GC-1 spg cells with or without 20 µg/ml of the purified recombinant Dpl for 0, 1, 2 or 4 h. Cell lysates were subjected to Western blots (Fig. 5B, a–c). The protein signals (Fig. 5B, a, b) were scanned, normalized to β-actin levels (Fig. 5B, c), and expressed as relative -fold changes over the signal in untreated GC-1 spg (Fig. 5B, a1) or N2a cells (Fig. 5B, b5, c5). Both in these two types of cells, levels of phosphorylated tumor suppressor p53 were relative low, *i.e.*, 1.00±0.05 and 0.90±0.08-fold, respectively (Fig. 5B, a1, a5, d). After incubation of N2a cells with Dpl, the level of the phosphrylated p53 at Ser15 was increased at 1 h (3.87±0.27-fold), 2 h (3.31±0.31-fold) and then drop down at 4 h (1.63±0.12-fold) (Fig. 5B, a6–8, d), indicating that the upstream active molecule, ATM might directly phosphorylate p53 [46] and p53 might be involved in Dpl-induced apoptosis. A downstream protein of p53, cyclin-dependent kinase inhibitor p21, was also elevated from 1.00±0.11-fold at 0 h to 4.02±0.39-fold at 1 h, 3.25±0.38-fold at 2 h and 1.47±0.15-fold at 4 h, respectively, with significant differences (*p*<0.01) (Fig. 5B, b6–8, e). p21 was further phosphorylated from 1.00±0.12-fold at 0 h to 20.29±3.10-fold at 1 h, 22.61±2.29-fold at 2 h and 8.66±1.01-fold at 4 h, respectively, with significant differences (*p*<0.01) (Fig. 5B, b6–8, f). These results suggest that (1) in unstressed N2a cells most p21 is present at a low level in an unphosphorylated form (Fig. 5B, b5, e), which is an indication that most p21 is localized in nuclei [47]; (2) followed by the responsive activation of p53 after Dpl stimulation (Fig. 5B, a6–8, d), the expression of p21 increased at 1 and 2 h and then dropped at 4 h (Fig. 5B, b6–8, e), indicating that elevation of p21 is p53 dependent; (3) little of phosphorylated p21 is present in unstressed N2a cells (Fig. 5B, b5, f), but Dpl stimulation caused the increase of the phosphorylated p21 (Fig. 5B, b6–8, f). The phosphorylation of p21 might happen at T145 (and S146) by the protein serine/threonine kinase (Akt) [48]. The phosphorylated p21 might relocalize from the nucleus to the cytosol and play its role in resisting Fas-mediated apoptosis [49]. Thus, Dpl-induced apoptosis in N2a cells might be through the ATM-p53-p21 pathway. It is notable that a low level of the phosphorylated p53 was present in GC-1 spg cells with or without incubation with Dpl, *i.e*., 1.00±0.05-fold at 0 h, 0.95±0.08-fold at 1 h, 1.11±0.10-fold at 2 h, and 1.08±0.09-fold at 4 h (*p*>0.05) (Fig. 5B, a1–4, d), indicating that Dpl could not increase the phosphorylated p53 level in GC-1 spg cells. Furthermore, undetectable unphosphorylated p21 (0-fold at 0, 1, 2 and 4 h) but abundant phosphorylated p21 is present in GC-1 spg cells before and after Dpl stimulation *i.e*., 50.36±5.6-fold at 0 h, 47.27±3.9-fold at 1 h, 47.76±5.04-fold at 2 h and 55.25±5.29-fold at 4 h, respectively, with no significant differences (*p*>0.05) (Fig. 5B, b1–4, f), suggesting that (1) unlike in N2a cells, the level of phosphorylated p21 in GC-1 spg cells is p53-independent; and (2) phosphorylated p21 in the cytosol of GC-1 spg cells might play roles in the control of apoptosis caused by Dpl.

### Dpl induces PrP^C^ elevation in N2a but not in GC-1 cells

Previous reports have shown an antagonistic interaction between Dpl and PrP^C^ [3], [4], [23], [31] and PrP^C^ elevation *via* ATM-mediated transcription pathway in response to copper-induced oxidative stress [44]. Here, we incubated N2a or GC-1 cells with Dpl (20 µg/ml) for 1 and 2 h and then detected PrP^C^ levels with mAb SAF-32 or SAF-70 (Fig, 1A, b). In the first hour, addition of Dpl to the N2a cells did not affect PrP^C^ expression level as similar PrP^C^ levels shown in cells with or without adding Dpl when PrP^C^ was detected with mAb SAF-32 (Fig. 5C, a6 vs. a5, b6 vs. b5). A much stronger protein intensity of PrP^C^ was detected 2 h after the treatment of Dpl than that without the treatment (Fig. 5C, a8 vs. a7, b8 vs. b7), indicating that the expression of PrP^C^ may be responsive to the Dpl stimulation. In contrast to the PrP^C^ elevation shown in the N2a cells, the N-terminal truncated PrP in the GC-1 spg cells did not appear to respond to Dpl (Fig. 5C, b1–4). Therefore, Dpl triggers the rapid elevation of the PrP^C^ protein in N2a cells but it somehow does not have the same inducing effect in GC-1 spg cells.

### Dpl increases ATM-dependent bindings of Sp1 and p53 to the *Prnp* promoter in N2a cells

**Figure 6:**
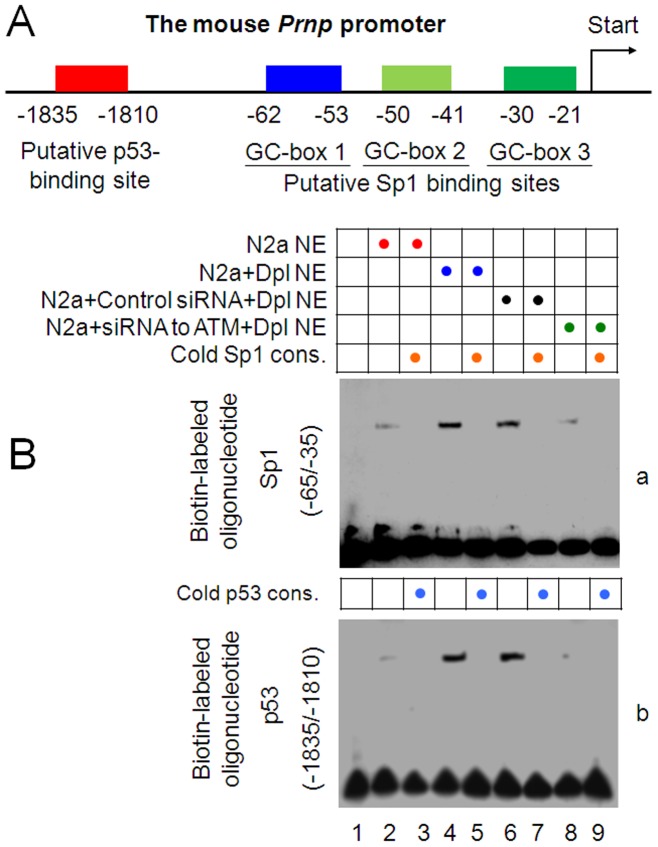
Dpl induces ATM-dependent bindings of Sp1 and p53 to the *Prnp* promoter. A. Schematic locations of the putative binding sites of Sp1 and p53 in the mouse *Prnp* promoter. B. ATM-dependent bindings of Sp1 and p53 to the *Prnp* promoter. N2a cells were mock- (lanes 2–5) or transfected with the control siRNA (lanes 6, 7) or siRNA to ATM (lanes 8, 9) for 48 h and then incubated with Dpl (20 µg/ml) for 2 h. Gel-shift analyses were then performed with nuclear extracts from cells. Combination of the oligonucleotides and the nuclear extract used in the assay are indicated by black circles above the radiogram.

After incubation of N2a cells with Dpl, the PrP^C^ expression is increased (Fig. 5C) accompanied with phosphorylation of p53 at ser15 (Fig. 5B), indicating that p53 may directly be phosphorylated by Dpl-activated ATM [46]. Therefore, we predicted that the activated ATM might activate p53 and initiate ERK/Sp1 pathways. The partial sequence of the mouse *Prnp* promoter has been analyzed, which reveals four motifs, three putative binding sites of Sp1 [32] and one of p53 (Fig. 6A). To clarify possible gene regulating mechanisms we performed EMSA using N2a cells to examine bindings of transcription factors, *i.e*., p53 and Sp1, to the mouse *Prnp* promoter. As target DNAs, the linear DNA fragments containing the putative Sp1 binding sites, GC-box 1 and 2 (−65/−35) and the putative p53 binding site (−1835/−1810) (Fig. 6A), were labeled with biotin at 3′-end. Incubation of biotin-labeled target DNA fragments with nuclear extracts from N2a cells induced shift bands (Fig. 6B, a2, b2). For identification of the binding nuclear proteins, 100-fold molar excess of the cold consensus oligonucleartides were added to the DNA/protein reactions. The cold p53 consensus [32] and the cold Sp1 consensus [33] were able to completely block the nuclear protein to the biotin-labeled *Prnp* putative p53 binding sequence (Fig. 6B, a3) and Sp1 binding sequence (Fig. 6B, b3), respectively, indicating that p53 and Sp1 do bind to the *Prnp* promoter. Incubation of cells with Dpl increased bindings of Sp1 and p53 to the *Prnp* promoter as DNA/protein binding bands were shown much stronger (Fig. 6B, a4–5, b4–5), indicating that Dpl enhances bindings of Sp1 and p53 to the *Prnd* promoter. To observe effects of ATM on binding of transcription factor to the *Prnp* promoter, we used siRNA to ATM to knock down the ATM expression in N2a cells. After mock-transfection or transfection with control siRNA or siRNA to ATM for 48 h, N2a cells were incubated with Dpl for 2 h and then nuclear proteins were extracted for EMSA. Introduction of the control siRNA did not affect binding patterns (Fig. 6B, a6–7, b6–7). Interestingly, knockdown of ATM by siRNA significantly reduced the signals of DNA/protein binding bands (Fig. 6B, a8–9, b8–9), indicating that Dpl-induced bindings of Sp1 and p53 to the *Prnd* promoter are ATM-dependent. Thus, ATM may be in a key position to upregulate the *Prnp* promoter after the Dpl stimulation. The responsive elevation of PrP^C^ to Dpl in N2a cells may be induced by ATM-mediated upregulation of the *Prnp* promoter, in which bindings of Sp1 and p53 to the promoter are enhanced.

## Discussion

As a prion-like protein, doppel has been showing its mystery since its discovery. Although the Dpl gene (*PRND* or *Prnd*) is downstream of the PrP gene (*PRNP* or *Prnp*), no detectable level of Dpl is expressed in the adult central nerve system. When *Prnd* was artificially activated, the PrP-knockout mice developed ataxia with Purkinje cell death [3]. In PrP^C^-depleted neuronal cells, such as mouse N2a and human SH-SY5Y cells, Dpl induces apoptosis [22], [31], [50]. Therefore, Dpl has been shown to be a toxic protein in the brain and neuronal cells. Unexpectedly, Dpl may be necessary in the male reproduction system evidenced by the Dpl-knockout experiments. The sperm from Dpl deficient mice appeared to be unable to undergo the normal acrosome reaction necessary to penetrate the zona pellucida of the oocyte [14], [15], [50]. Simply incubation of ram spermatozoa with the recombinant ovine Dpl polypeptide during the capacitation process significantly improved the sperm motility and vigour and may enhance *in vitro* spermatozoa fertilizing ability [26]. Previous reports and our experiments have demonstrated that Dpl is not expressed in the adult brain and cerebellum but in epididymis, testis and spermatozoa, suggesting that Dpl might play some roles in the male reproduction system. Therefore, it is necessary to understand the differentially molecular activities when neuronal and spermatogenic cells expose to Dpl.

Even though Dpl expression patterns have been found opposite in neuronal and spermatogenic cells [3], [4], [51], the molecular mechanism underlying the difference is not well known. There are strong indications suggesting that the level of the Dpl expression is regulated at the transcriptional level. For example, knockout of a transcriptional factor Brn-3a in mice dramatically decreased in the Dpl expression in the embryonic neurons of dorsal root ganglia and spinal cord that typically had relatively high levels of the Dpl protein [28]. Consistently, introduction of the neuroblastoma ND7 cells, that normally express low level of Dpl, with Brn-3a increased the *Prnd* mRNA expression [28], suggesting that Brn-3a is specifically involved in the positive transcriptional regulation of the *Prnd* gene expression. In most of the prior Dpl studies, the *Prnd* expression was artificially elevated in the brain of transgenic mice by fusion of the highly expressing *Prnp* promoter to the *Prnd* gene [3], [20], [52], further confirming transcriptional repression of the *Prnd* gene in the mouse brain. By adapting an *in vitro* report system we have shown that the *Prnd* transcription level is much higher in GC-1 spg cells than that in N2a cells (Fig. 2). Therefore, different sets of positive and negative transcription factors might regulate the *Prnd* expression in GC-1 spg and N2a cells. By using the electrophoretic mobility shift assay (EMSA), we have identified the different binding patterns between transcription factors and the *Prnd* promoter in these two types of cells. First, the upstream stimulatory factors (USF) binds to E-box both in GC-1 spg and N2a cells; second, the positive transcription factor, Brn-3 [28], binds to the *Prnd* promoter in GC-1 spg but not in N2a cells; third, Sp1, a transcriptional activator [36], [37], binds to GC-box in GC-1 spg cells and Sp3, a transcriptional repressor or activator [38], binds to GC-box in N2a cells (Fig. 3). Our findings may provide an explanation that the high and low level of Dpl in GC-1 spg and N2a cells may be regulated by binding of different transcription factors to the *Prnd* promoter.

Dpl is not significantly present in the brain and neuronal cells, such as N2a cells, but abundantly expressed in testis and spermatogenic cells, such as GC-1 spg cells (Fig. 1), indicating that the principally physiological function of Dpl is not in the central nerve system but in the male reproduction system. Dpl induces ROS accumulation, cell death and apoptosis in N2a but not in GC-1 spg cells (Fig. 4, 5), indicating that the response of these two cell lines to Dpl effects in opposite fashions. Previous studies have shown that different types of cells may differ in their response to the metal stress induced by Cu(II) *via* activation of the tumor suppressor p53 and the cyclin-dependent kinase inhibitor p21(Cip1/WAF1/Sdi1) (p21), which limits proliferation by cell cycle arrest [31], [53], [54]. Indeed, similar to prior studies [38], [55], we have demonstrated that, in N2a cells, Dpl induces oxidative stress, following activation of p53 that mediates the elevation of un-phosphorylated p21 and finally results in apoptosis (Fig. 4, 5A, B). On the other side, in GC-1 spg cells the abundant phosphorylated p21 is present and additional Dpl does not change the phosphorylation status of p21 (Fig. 5B). This is a sign of anti-apoptosis in GC-1 spg cells [56], [57]. Therefore, our findings indicate that GC-1 spg cells are resistant to apoptosis induced by Dpl and N2a cells are prone to Dpl-induced apoptosis.

Although the full-length PrP^C^ is expressed in brain and testis, only N-terminally truncated PrP is present in mature spermatozoa [15], indicating possibly different functions of PrP in neuronal and spermatogenic systems. Indeed, the incubation of N2a cells with Dpl increases the ATM-modulating bindings of transcription factors Sp1 and p53 to the *Prnp* promoter (Fig. 6), and then induces the elevation of the full-length PrP^C^ (Fig, 5C) that might contribute in counteraction against the Dpl toxicity, similar to the function of PrP^C^ in response to Cu(II)-induced oxidative stress [44]. Interestingly, PrP in the GC-1 cells appears to miss the N-terminal end and does not response to the Dpl exposure (Fig. 5C). Therefore, the truncated PrP might have a parallel effect as Dpl in gametogenesis.

In conclusion, our data strongly indicate the differential responses of pro-apoptotic (neuronal N2a) and anti-apoptotic (GC-1 spg) cells to the Dpl toxicity. The low and high levels of Dpl in N2a and GC-1 spg cells are transcriptionally regulated. The transcription factor USF binds to the *Prnd* promoter both in two types of cells, the transcription activators Brn-3 and Sp1 only in GC-1 spg cells, and the potential transcription suppressor Sp3 only in N2a cells. Dpl induces oxidative stress and apoptosis in N2a cells, which causes the ATM-modulating bindings of transcription factors Sp1 and p53 to the *Prnp* promoter, resulting in the PrP^C^ elevation for counteraction against the Dpl cytotoxicity. GC-1 spg cells does not response to Dpl effects. The N-terminal truncated PrP might have a parallel effect as Dpl in gametogenesis.
